# Supplementary material for: Mental Health Literacy about Personality Disorders: A Multicultural Study
Source: Behav Sci (Basel). 2023 Jul 21;13(7):605. doi: 10.3390/bs13070605 (PMC10376649; doi:10.3390/bs13070605)
Supplement: Supplementary file 1 [file behavsci-13-00605-s001.zip › behavsci-2277548-supplementary.pdf]

Supplementary Materials to

Mental Health Literacy on Personality Disorders: A Multicultural Study

Kerim Alp Altuncu [1], Arianna Schiano Lomoriello[2], Gabriele Lo Buglio[3], Ludovica Martino [4], Asrin Yenihayat[5], Maria Teresa Belfiore[4], and Tommaso Boldrini[4,*]

1 Department of General Psychology, University of Padova;
2 Department of Cognitive System, Denmark Technical University (DTU), Copenhagen, Denmark;
3 Department of Dynamic and Clinical Psychology, and Health Studies, Faculty of Medicine and Psychology, Sapienza University of Rome;
4 Department of Developmental Psychology and Socialization, University of Padova;
5 Department of Clinical Psychology, Psychology for Individuals, Families and Organizations. Faculty of Human Sciences, University of Bergamo.

* Correspondence: tommaso.boldrini@unipd.it.

Index

S1. An example of the survey vignettes and questions asked to participants

S2. Ranking of labels given by respondents, grouped into categories

S1. An example of the survey vignettes and questions asked to participants

Survey vignette:

Barry is a single 45-year-old man working in a post office. He enjoyed this job as it involved little contact with others. He refused several promotions because he feared the social pressures. He supervises a number of employees but still finds it hard to give instructions even to people he has known for years. Barry had dated a few women he met through family introductions. He was never confident enough to approach a woman on his own. Perhaps it was his shyness that first attracted Steph, his co-worker. Steph had asked him out, but Barry declined at first, claiming some excuse. When Steph asked again a week later, Barry agreed thinking she must really like him if she were willing to pursue him. The relationship developed and soon they were dating every night. However, the relationship strained when Barry interpreted any slight hesitation in her voice as a lack of interest. He repeatedly requested reassurance that she cared for him and evaluated every word and gesture for evidence of her feelings. When Steph said she could not see him because she was tired. he assumed she was rejecting him. After several months, the relationship ended because Steph could not stand Barry's constant nagging. Barry assumed that Steph had never really cared for him.

Survey questions:

In general, how happy do you think Barry is?

Very 5 4 3 2 1 not at all

In general, how successful at his work do you think Barry is?

Very 5 4 3 2 1 not at all

In general, how satisfying do you think Barry's personal relationships are?

Very 5 4 3 2 1 not at all

Do you think that, in any sense they have a psychological problem?

Very 5 4 3 2 1 not at all

If so, what is it? ……..

(open ended question)

S2. Ranking of labels given by respondents, grouped into categories

a Ranking of labels for schizotypal personality disorder

Label category	Answer, %
None	51.1
Other / Non-specific[i]	25.2
Denial of reality	8.8
Schizophrenia	8.4
Loneliness	6.5
Insecurity/lack of confidence	6.1
Depression	5.8
Loss of/Dependence to mother	4.5
Anxiety/fear	3.4
Paranoia	3.1
Asocial	2.7
Other personality disorders	2.3
Schizoid	1.5
I don't know	1.1
Schizotypal	0.8

[i]Examples: ‘longing’, ‘in mother’s womb’

b Ranking of labels for paranoid personality disorder

Label category	Participants, %

None	48.9
Trust issues	18.3
Skepticism/sceptical	12.2
Other/non-specific	11.1
Paranoia/paranoid	9.9
Insecurity/lack of confidence	4.6
Paranoid personality disorder	2.7
Schizophrenia	1.2
I don’t know	0.8

[i]Examples: ‘spiritual problems’, ‘immature personality’

c Ranking of labels for antisocial personality disorder

Label category	Participants, %
None	48.9
Other / non-specific	20.6
Family dysfunction trauma/father-mother issue	11.9
Lying/mythomania	6.9
Rebelliousness/lack of authority	4.6
Sociopath/psychopath	3.1
Insecurity/lack of confidence	3.1
I don’t know	2.7
Violence/anger	2.7

Loneliness	2.3
Behavioral disease	2.3
Antisocial personality disorder	1.9
Depression	1.9
Unspecified personality disorder	1.9
Criminal disposition	1.6
Anti-social	1.5

[i]Examples: ‘piece of shit’, ‘adaptation’

d Ranking of labels for borderline personality disorder

Label category	Participants, %
Other / non-specific	29.3
None	26.3
Bipolar	12.3
Unbalanced/unhappy	7.7
Other/unspecified personality disorders	7.7
Depression	7.2
Insecurity/lack of confidence	5.4
Borderline personality disorder	4.6
Anger/violence	3.4
Trust issues	1.9
Trauma	1.9

I don’t know	1.5

[i]Examples: ‘thoughtless’, ‘personality breakdown’

e Ranking of labels for narcissistic personality disorder

Label category	Participants, %
None	32.4
Narcissism/narcissistic	19.5
High ego/self-centeredness/megalomaniac	13.4
Selfishness / egoist	11.1
Other/non-specific	11.1
Arrogance/vanity	5.3
Approval/attention- seeking	5.0
Narcissistic personality disorder	3.4
Unspecified/other PD	1.9
I don’t know	1.5

[i]Examples: ‘loser mentality’, ‘pessimism’

f Ranking of labels for avoidant personality disorder

Label category	Participants, %
Insecurity/low self-esteem	35.1
None	24
Anxiety/anxiety disorder/social anxiety	11.8

Other/non-specific[i]	11.1
Communicational/relational problems	6.5
Trust issues	6.5
Asocial	4.2
Sceptical/paranoid	3.9
Introversion/shyness	3.8
Trauma	2.7
Unspecified personality disorder	2.3
Antisocial	1.1
I don't know	1.1

[i]Examples: 'needs therapy', 'complex'

g Ranking of labels for obsessive-compulsive personality disorder

Label category	Participants, %
None	35.9
Perfectionism/perfectionist	21.4
Other/non-specific[i]	14.5
Control freak/control issues	9.2
Obsessive-compulsive	6.5
Obsessed/obsessive/obsession	4.6
Workaholic	3.8
Anxiety	2.7

Insecurity	2.7
Obsessive-compulsive personality disorder	1.9
I don't know	1.9

[i]Examples: 'knows all', 'caring too much for little things'
